# Microbe Profile: Pseudonocardia: antibiotics for every niche

**DOI:** 10.1099/mic.0.001501

**Published:** 2024-09-19

**Authors:** Bonnie Whatmough, Neil A. Holmes, Barrie Wilkinson, Matthew I. Hutchings, Jonathan Parra, Katherine R. Duncan

**Affiliations:** 1 Department of Molecular Microbiology, John Innes Centre, Norwich Research Park, Norwich, NR4 7UH, UK; 2 Instituto de Investigaciones Farmacéuticas (INIFAR) and Centro de Investigaciones en Productos Naturales (CIPRONA), Universidad de Costa Rica, San José 11501-2060, Costa Rica; 3 Centro Nacional de Innovaciones Biotecnológicas (CENIBiot), CeNAT-CONARE, San José 1174-1200, Costa Rica; 4 Newcastle University, University of Newcastle Biosciences Institute, Newcastle Upon Tyne, NE2 4HH, UK

**Keywords:** Actinomycetes, natural products, *Pseudonocardia*, specialised metabolites

## Abstract

*Pseudonocardia* species comprise a genus of filamentous, sporulating bacteria belonging to the phylum Actinomycetota, formerly Actinobacteria. They are found in marine and freshwater sediments and soils and associated with marine animals, insects, and plants. To date, they have mostly been studied because of their mutually beneficial symbiosis with fungus-growing ants in the tribe *Attini*. They have also attracted interest due to their biosynthetic capabilities, including the production of variably glycosylated polyenes and other novel antifungal compounds, and for their capacity to grow on a variety of hydrocarbons. The majority of clinically used antibiotics are derived from the specialised metabolites of filamentous actinomycete bacteria and most of these come from the genus *Streptomyces*. However, in the quest for novel chemistry there is increasing interest in studying other filamentous actinomycete genera, including *Pseudonocardia*. Here we outline the biological properties, genome size and structure and key features of the genus *Pseudonocardia*, namely their specialised metabolites and ecological roles.

## Graphical abstract

Scanning electron microscopy (SEM) images of *Pseudonocardia* sp. (a). Ae707-Ps1 and (b). Ae707-Ps2. These are the two lineages of *Pseudonocardia* associated with *Acromyrmex echinatior* leafcutter ants collected in Gamboa, Panama. (c). Four specialised metabolites are shown that are produced by *Pseudonocardia* strains isolated from diverse ecosystems: they are 9-methoxyrebeccamycin, selvamicin, pseudonochelin and attinimicin. (d). This tree shows a subset of phylogenetic diversity showing strain origin (outer ring colour), genome size (Mb, red box) and number of biosynthetic gene clusters (BGCs, purple box). The specialised metabolites (c) are coloured to match the producing strains, the boxed numbers show the genome size (red) and number of BGCs (purple) (d).

## Taxonomy

Phylum: Actinomycetota; class: Actinomycetes; order: Pseudonocardiales; family: *Pseudonocardiaceae;* type genus: *Pseudonocardia*.

## Properties

*Pseudonocardia* spp. are aerobic, slow-growing, non-motile Gram-positive bacteria. Typically, strains form branched substrate hyphae that may fragment into rod-shaped elements. Aerial hyphae, if formed, can fragment into chains of oval or square elements, or differentiate into chains of spores. Substrate and aerial hyphae undergo cell division in several different directions simultaneously, with a tendency to form swellings. Spores are usually smooth and may be formed on the substrate or aerial hyphae. The major menaquinone is MK-8(H_4_) and the predominant fatty acid is *iso*-branched hexadecanoic acid, while mycolic acids are absent from the cell wall.

Colonies form pigmented orange substrate hyphae on solid agar followed by the formation of reproductive white aerial hyphae that undergo cell division to form chains of spores [1]. They are typically very slow growing on agar and many *Pseudonocardia* strains do not grow at all in liquid culture. The size and appearance of colonies varies greatly between strains and depending on the growth medium used. Strains have been isolated from marine sediment using A1 and SW (seawater) agar supplemented with sea salt, and ant-associated strains have been isolated using soya flour mannitol agar (SFM). Like other filamentous actinomycetes, their colonies have a hairy or fuzzy appearance, but they are usually smaller than *Streptomyces* and *Amycolatopsis* species. Their circular genomes contain multiple biosynthetic gene clusters (BGCs) encoding for enzymes involved in the biosynthesis of specialised metabolites [2], including antibiotics, and they make glycosylated variants of nystatin, which have attracted interest due to their increased solubility in water. *Pseudonocardia* have to this point remained challenging in terms of genetic manipulation, with many strains determined to be recalcitrant to any form of genetic transformation . To our knowledge, there are no published reports of genome editing using CRISPR–Cas systems in *Pseudonocardia* species and the strains we have worked with do not maintain any of the self-replicating plasmids commonly used for *Streptomyces* research. However, genes have been deleted using homologous recombination, including PCR-targeted cosmids and suicide vectors. Genes can also be over-expressed by integrating plasmids such as pSET152 into the phage phiC31 attachment site [3]. Biochemical tests that can be used to discriminate between *Pseudonocardia* include those for starch hydrolysis, oxidase, catalase, nitrate reductase, urease, acid production of carbohydrates and gelatinase, though these methods are unlikely to be as effective as molecular identification through whole-genome sequencing [4].

## Genomes

The first *Pseudonocardia* genome, of the 1,4-dioxane-degrader *Pseudonocardia dioxanivorans* CB1190, was completed in 2011. Since then, more than 180 *Pseudonocardia* genome sequences have been published, and these include 11 complete genomes, 1 chromosome, 52 scaffold genomes (1 or more contigs are connected across gaps of 10 or more bases to create scaffolds), and 121 contig genomes (sequences do not contain gaps and are unplaced or unlocalised). *Pseudonocardia* bacteria typically contain circular chromosomes, in the range of 6–7 Mbp, and some strains also carry plasmids. An exception to this is *Pseudonocardia* sp. DSM 110487, which has a chromosome >10 Mbp. The GC content of their DNA is >70% and their genomes typically contain 4500–7500 open reading frames (ORFs) (>9000 in *Pseudonocardia* DSM 110487) and 50–85 putative non-coding RNAs. The publicly available genomes contain between 10–30 specialised metabolite BGCs [5] and some *Pseudonocardia* strains also carry BGCs on plasmids, which likely mediate horizontal gene transfer between strains. Despite their importance as symbionts, there is no evidence of genome degradation.

## Phylogeny

The genus *Pseudonocardia* was first described in 1957 and at the time of writing, 68 species of *Pseudonocardia* are listed in the LPSN (List of Prokaryotic names with Standing in Nomenclature), although most do not have genome sequences and, as such, the phylogenetic relationships are largely unknown, as 16S rRNA gene sequences are unreliable for actinomycetes. They are found in diverse environmental niches, suggesting they have not evolved to a specific host. However, this assumption is challenged by their established role as defensive mutualists in attine fungus-growing ant systems where they produce antimicrobials to shape the cuticular microbiome and combat fungal parasites [6,7]. Intriguingly, symbiotic *Pseudonocardia* spp., including attine ant-associated strains, do not form distinct clades within the genus but instead group together with environmental strains [7].

## Key features and discoveries

*Pseudonocardia* species have been isolated from soils, freshwater and marine sediments, marine sponges, corals, attine fungus-growing ants, and from the phyllosphere, roots and rhizosphere of plants [8]. The best-characterised *Pseudonocardia s*trains are defensive mutualists of fungus-growing ants of the tribe *Attini* in which individual ant colonies house and feed a single strain of *Pseudonocardia* bacteria on the surface of their exoskeletons [9]. These mutualist strains are transmitted vertically and the *Pseudonocardia* bacteria have a dual function: they prevent other bacteria from colonising the ant cuticles [4] and they provide the ants with antifungal compounds to combat fungal pests such as *Escovopsis* spp., which parasitise their fungal cultivar [5,7, 9]. Several new antifungal compounds have been isolated from *Pseudonocardia* mutualist strains, including unusual polyenes, the nonribosomal peptide attinimicin, and the depsipeptide dentigerumycin, suggesting that they remain a promising source of new chemistry. These strains also produce broad-spectrum antibacterials that inhibit a wide range of Gram-positive and Gram-negative bacteria [7]. Free-living *Pseudonocardia* strains have also been the source of broad-spectrum antibacterials, including branimycins, made by *Pseudonocardia carboxydivorans* M-227. Many other strains have been reported to have anti-viral, anti-tumour, anti-hyperglycaemic, neuroprotective, plant growth-promoting and inhibitive of mycotoxin production, indicating the range of bioactive potential. In several strains, observed bioactivity is yet to be linked definitively to a specialised metabolite and BGC, and, furthermore, a large majority of BGCs encoded within *Pseudonocardia* genomes are of unknown function. *Pseudonocardia* species have also been reported to degrade environmental contaminants that are harmful to humans, including alkylpyridines which contaminate groundwater, 1,4-dioxanes which contaminate drinking water and tetrahydrofurans plus other toxic precursors such as polylactic acid which are widely used to make plastics. Thus, some *Pseudonocardia* species have been proposed as potential bioremediation strains, although their slow-growing nature may adversely affect their utility [4].

## Open questions

- Why did fungus-growing attine ants choose these slow-growing bacteria as mutualists?
- Why are symbiont genomes not reduced relative to free-living strains?
- Are they also defensive mutualists of plants, sponges and corals?
- What metabolites do they make to inhibit Gram-positive and Gram-negative bacteria?
- Why are they resistant to genetic manipulation?
- What is the ecological role of their specialised metabolites?

## Abbreviations

BGC: Biosynthetic Gene Cluster
CRISPR: Clustered Regularly Interspaced Short Palindromic Repeats
LPSN: List of Prokaryotic names with Standing in Nomenclature
SEM: Scanning Electron Microscopy
